# Flow Cytometric Single-Cell Identification of Populations in Synthetic Bacterial Communities

**DOI:** 10.1371/journal.pone.0169754

**Published:** 2017-01-25

**Authors:** Peter Rubbens, Ruben Props, Nico Boon, Willem Waegeman

**Affiliations:** 1 KERMIT, Department of Mathematical Modelling, Statistics and Bioinformatics, Ghent University, Ghent, Belgium; 2 Center for Microbial Technology and Ecology (CMET), Ghent University, Ghent, Belgium; Argonne National Laboratory, UNITED STATES

## Abstract

Bacterial cells can be characterized in terms of their cell properties using flow cytometry. Flow cytometry is able to deliver multiparametric measurements of up to 50,000 cells per second. However, there has not yet been a thorough survey concerning the identification of the population to which bacterial single cells belong based on flow cytometry data. This paper not only aims to assess the quality of flow cytometry data when measuring bacterial populations, but also suggests an alternative approach for analyzing synthetic microbial communities. We created so-called *in silico communities*, which allow us to explore the possibilities of bacterial flow cytometry data using supervised machine learning techniques. We can identify single cells with an accuracy >90% for more than half of the communities consisting out of two bacterial populations. In order to assess to what extent an in silico community is representative for its synthetic counterpart, we created so-called *abundance gradients*, a combination of synthetic (i.e., in vitro) communities containing two bacterial populations in varying abundances. By showing that we are able to retrieve an abundance gradient using a combination of in silico communities and supervised machine learning techniques, we argue that in silico communities form a viable representation for synthetic bacterial communities, opening up new opportunities for the analysis of synthetic communities and bacterial flow cytometry data in general.

## Introduction

Microbial communities are primary contributors in most biogeochemical processes on Earth [1]. As such, a large portion of microbial research has been dedicated to the study of the structure and functionality within microbial communities of various complexities. Historically, these aspects have been largely inferred from research with axenic cultures. Nowadays, the availability of next-generation sequencing technologies has shifted the focus towards the study of microbial taxa directly in their respective environment (’omics). However, both approaches suffer from either a lack of complexity (axenic cultures) or a lack of controllability (’omics) [2].

To cope with these bottlenecks, synthetic microbial communities, assembled through the selection of individual microbial populations, and studied under controlled environmental conditions, have recently been suggested as promising intermediary platforms [2–5]. Advanced cultivation methods have allowed researchers to construct defined and diverse synthetic bacterial consortia for both ecological and biotechnological research [6]. Depending on the goal of the study, these synthetic bacterial consortia may consist out of several [5, 7] to more than ten taxa [8, 9]. The goals of these studies can be manifold; on the one hand, enhanced biotechnological conversion processes such as production of biofuels are envisioned [10, 11], while on the other hand synthetic communities are used as simplified ecosystem models for developing ecological theories [7, 8]. It is worth noting that the latter studies have facilitated advanced experimental design, with large microcosm studies using more than thousands of consortia.

With the number of synthetic ecology studies ever increasing, the analysis of low-complexity community compositions, i.e. quantifying the abundance of each constituent taxon, remains the most significant challenge. A study by Saleem et al. used traditional plate counting, which entailed the cultivation of all individual members on agar plates followed by subsequent enumeration of the colony forming units (CFU) [7]. Their counting approach benefited from the fact that each microbial population in their study had distinct morphological characteristics. However, cultivation-based enumeration inevitably suffers from a significant source of bias, since lab cultures frequently adopt a viable but non-culturable state (VBNC) [12]. This results in inflated numbers of false negative counts, and as such, to severe underestimations of population densities.

Other studies, such as the one by Mee et al., applied quantitative PCR (qPCR) [9]. Yet, while successful for their *Escherichia coli* mutants, the analysis of complex synthetic communities that consist of diverse taxa (e.g., mixtures of Gram-positive and Gram-negative bacteria) faces considerable bias due to taxon-dependent nucleic acid extraction efficiencies, varying amplification efficiency and also primer selectivity. In extremis, this has limited studies with complex synthetic communities to relate their temporal observations only to the initial community composition [8]. Overall, there exists a lack of streamlined and validated methods to monitor the composition of synthetic consortia.

Flow cytometry (FCM) offers a multiparametric description of individual cells, which can be applied to study microbial communities [13, 14]. As the speed of measurement is increasing (up to 50,000 of cells per second), alongside with the dimensionality of the data, the number of computational and statistical methods and applications, shortly dubbed as FCM *bioinformatics*, is growing accordingly [15].

The main goal of this paper is to explore in a systematic way the possibilities of using FCM data to identify bacterial single cells, in order to be able to characterize the composition of synthetic bacterial communities. We will do this by introducing the concept of *in silico communities*. These are communities created by an aggregation of FCM data coming from axenic cultures which are being measured separately through FCM. The great advantage of using this approach is that we know which cell stems from which bacterial population. This enables us to apply a supervised machine learning approach, which has shown previous success in the recognition of leukemia [16] or to find markers which are able to discriminate between tumor and normal cells in lung cancer [17]. More specifically, artificial neural networks have been used to identify various populations of phytoplankton [18, 19]. Applied to bacterial populations, this approach has been used to analyze the effect of various cocktails of fluorescent staining [20] or to analyze the extent to which individual cells can be classified using multiple scatter signals [21]. However, the number of populations used in these latter studies is small, studying only pairwise combinations of two taxa.

In the first part of the paper we analyze to what extent data coming from FCM can be used in order to separate microbial populations at the single-cell level. To do so we have cultivated twenty axenic cultures and characterized them by FCM. We performed single-cell predictions using Linear Discriminant Analysis (LDA), an established method for performing multivariate analyses in microbial ecology [22], and a Random Forest classifier, a robust classifier known for its high performance in various applications [23].

In the second part we show to which degree an in silico community is able to identify a synthetic bacterial community. This is not a foregone conclusion due to the heterogeneous character of bacterial populations, which is reflected in FCM data [24]. In order to do so we created so-called *abundance gradients*, a combination of in vitro communities which consist out of two populations in varying abundances. We will show that we are able to retrieve these relative abundances, using a classifier trained on an in silico community; this result enables researchers to perform a supervised analysis of synthetic microbial communities.

**Fig 1:**
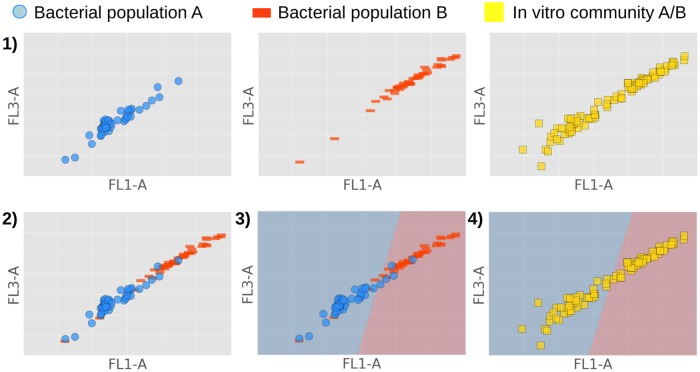
Proposed method to identify the composition of a synthetic microbial community comprising bacterial populations A & B. **1)** Measure both bacterial populations A & B separately through FCM, as well as the synthetic community made up out of A & B. **2)** Create in silico community by aggregating datafiles of individual populations. **3)** Choose classifier and train it on silico community to learn decision boundary (evaluated in the first part of the paper). **4)** Use trained classifier to identify the composition of a synthetic bacterial community (evaluated in the second part of the paper).

In the third part of the paper we estimate to what extent bacterial communities can be analyzed for higher population complexities, i.e., in a *multiclass* setting. To do so we created and evaluated in silico communities containing more than two populations. The results show that our approach is valid for communities of lower complexities, furthermore FCM gives rise to data that should be feasible for higher complexities as well. A schematic overview of the proposed method can be found in Fig 1.

## Results

### Classification performances on binary in silico communities

The performances using LDA and a Random Forest classifier were calculated for all possible pairwise combinations considering twenty populations for *S* = 2, *S* denoting the number of populations making up a community, i.e., the *population richness*. This results in 190 in silico communities, where the same amount of cells was sampled for each population, thus creating evenly distributed in silico communities. We calculated the mean for the area under the ROC curve (AUC) and the accuracy (acc), accompanied with their standard deviations and the percentage of communities which reported a score higher than 0.90; results are reported in Table 1.

**Table 1:** Performances using LDA and Random Forests (RF) for *S* = 2. Both classifiers were trained on 70% of the data for all 190 in silico communities, after which they predicted the population to which individual cells belong contained in 30% held-out test sets. We denote the mean AUC (*μ*_AUC_) and accuracy (*μ*_acc_), along with their standard deviation (*σ*_AUC/acc_) and the percentage of communities reporting a performance of 0.90 or higher.

	*μ*_AUC_	*σ*_AUC_	AUC > 0.90	*μ*_acc_	*σ*_acc_	acc > 0.90
LDA	0.90	0.089	62%	0.83	0.088	27%
RF	0.95	0.071	82%	0.90	0.085	65%

**Fig 2:**
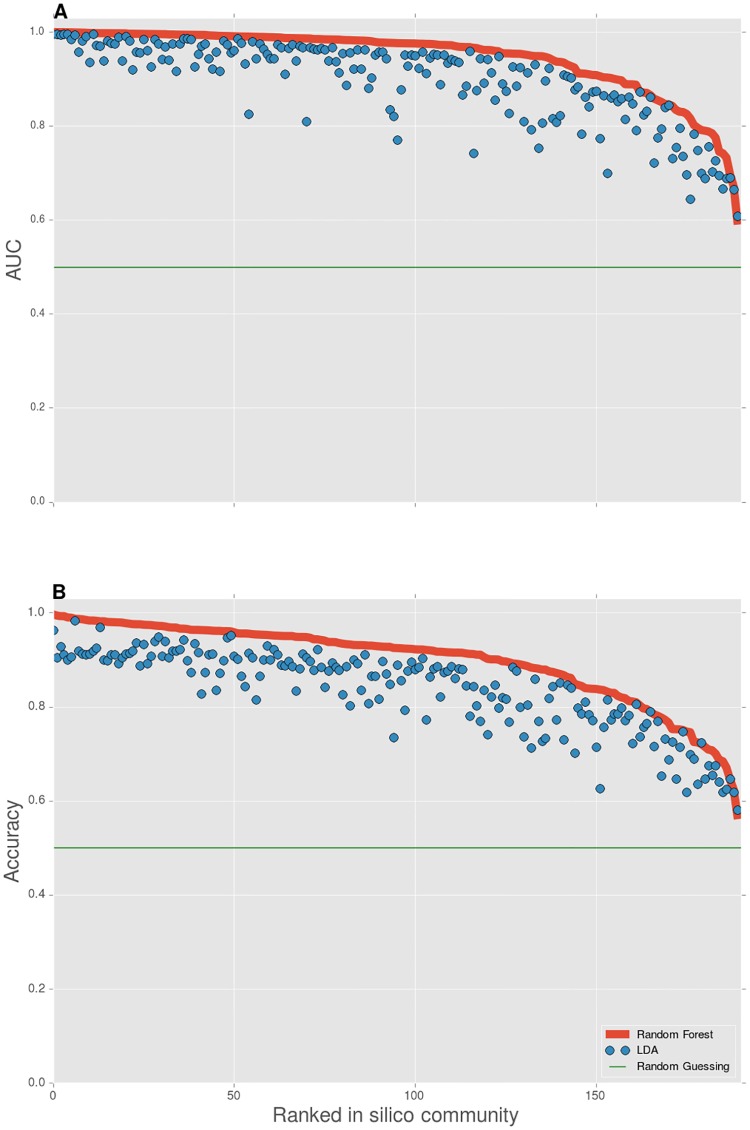
Classifier performances using LDA and Random Forests for *S* = 2. **A** AUC. **B** Accuracy. Performances are visualized for all 190 evenly distributed in silico communities; the performances have been calculated on a 30% held-out test set. The in silico communities have been ranked in descending order according to the performances resulting from using Random Forests, accompanied with performances resulting from LDA on the same in silico community.

We conclude that for a majority of in silico communities we are able to perform single-cell predictions up to high performances, especially when using Random Forests; in this case more than half of our communities report results higher than 0.90 for both AUC and the accuracy. Our highest performances top off at an AUC of 0.999 and an accuracy of 0.996. To further illustrate our findings we have visualized the AUC and the accuracy for all in silico communities, where performances have been ranked in descending order according to the results of applying a Random Forest classifier (Fig 2).

On average we see that a Random Forest classifier performs better than LDA. However, it is not always necessary to use a ‘black-box’ non-linear classifier such as a Random Forest. For some of the in silico communities we see that the performance of LDA is similar to the performance of Random Forests; 45% of the in silico communities report an increase in AUC of less than 0.03, 17% report an increase in accuracy less than 0.03. Moreover, note that pairwise combinations of populations give rise to performance accuracies ranging from 99% to near random guessing predictions. Hence, our dataset is highly representative, that is, we were not biased towards highly discriminative populations.

### Predicting the abundance gradient

**Fig 3:**
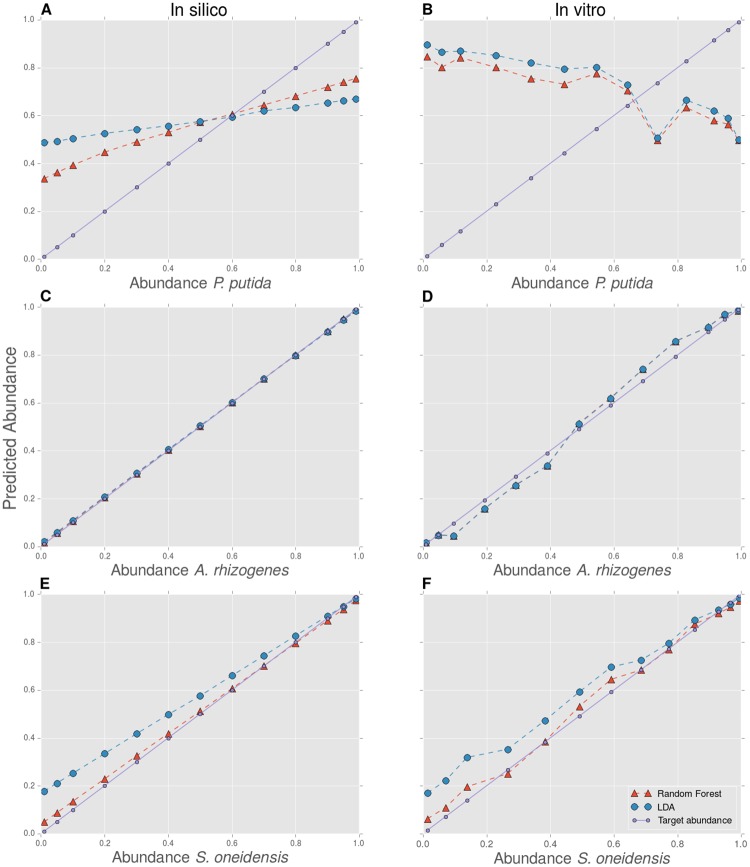
Predicted abundance gradients. **AB** Comb. 1: *P. putida—P. fluorescens*; **CD** Comb. 2: *A. rhizogenes—Janthinobacterium sp. B3*; **EF** Comb. 3: *S. oneidensis—M. luteus*. Both the in silico (left panel) and in vitro (right panel) constructed abundance gradients are visualized. The predicted relative abundance gradients is plotted against its target relative abundance (designed in silico and in vitro) for the first bacterial population of the three combinations. The relative abundance of the opposite population equals one minus the relative abundance of the first population (as *S* = 2).

An abundance gradient consists out of a set of bacterial communities containing two populations in varying abundances. We constructed these gradients for three combinations of bacterial populations, combinations for which we initially reported a low (Comb. 1), medium (Comb. 2) and high performance (Comb. 3) respectively. We created these gradients in vitro, but, because we measured the bacterial cultures separately beforehand through FCM, we were also able to construct these gradients in silico. In order to explore to what extent in silico communities can be used to identify synthetic bacterial communities, we have predicted the relative abundances of both in silico and in vitro abundance gradients, using LDA and a Random Forest classifier trained on a full evenly distributed in silico community. Ideally, a classifier which is able to achieve a high AUC and accuracy on a held-out test set of this in silico community gives rise to a well-predicted abundance gradient, both in silico and in vitro. The predicted abundance gradients are visualized in Fig 3.

As expected, the predicted abundance gradients for Comb. 2 and 3 match the target abundance gradients (Fig 3C, 3D, 3E and 3F), whereas this is not the case for Comb. 1 (Fig 3A and 3B). We highlight the similar behavior for the in silico gradient (left panel) and the in vitro gradient (right panel). First, we note a systematic bias using LDA for Comb. 3; although trained on an evenly distributed in silico community, the classifier systematically favors the *S. oneidensis* population.

Second, we note that for Comb. 2 the predicted gradients highly overlap; this means that an analysis using LDA and a Random Forest gives rise to very similar results. This is however not the case for Comb. 3, where the use of Random Forests results in a gradient that lies closer to the target gradient, which is reflected for both the in silico and the in vitro analysis. These observations are reflected in the root mean squared error (RMSE), which is calculated between the predicted gradients and the known target abundance gradients (Table 2). The RMSE for the in silico analysis can be interpreted as the most optimal value to achieve for a classifier when analyzing an in vitro community. We see that the RMSE gives comparable results when performing an in silico or in vitro analysis for Comb. 3, this is however not the case for Comb. 1 or 2. This can result from experimental noise when creating in vitro gradients.

**Table 2:** RMSE for predicted abundance gradients. RMSE has been calculated between the predicted gradients and the target gradients, both in silico and in vitro, having used LDA and a Random Forest classifier.

	Comb. 1	Comb. 2	Comb. 3
RMSE LDA in silico	0.29	0.0060	0.10
RMSE LDA in vitro	0.51	0.036	0.096
RMSE RF in silico	0.21	0.0036	0.022
RMSE RF in vitro	0.48	0.036	0.032

Using the knowledge that FCM analyses generally do not exceed a 5% instrumental error [25], we performed a comparable analysis in terms of the Hill number of order one, i.e., the exponential of the *Shannon diversity*, noted as *D*_1_ [26]; this diversity index gives information concerning the *evenness* of a community (see Appendix: Alpha diversity analysis). Because mathematical properties of this index allow us to combine uncertainties for all relative abundances characterizing a microbial community, we can calculate confidence intervals (CI) within which our in vitro target abundance gradient should lie. Inspecting the results, we see that our predicted abundance gradients for most communities in Comb. 2 (both LDA and Random Forests) and 3 (Random Forests) lie within the 68%-CI; all of them lie within the 95%-CI (S4 Fig).

The results for the in vitro analysis of Comb. 2 and 3 are similar, although we would expect from initial performances that these values would be different. To investigate this issue, we added additional results in Table 3, for which we report the performance of a classifier on a held-out test set of the new in silico communities in terms of the accuracy and the AUC, compared to the original values calculated in the previous section (*). In order to be able to make a comparison, classifiers were trained and evaluated in exactly the same way.

**Table 3:** Performance comparison for the in silico communities that are present in both dataset 1 and 2. Classifier performance comparison on a held-out test set for dataset 1 (denoted with *) and 2 for those in silico communities that are present in both datasets. These in silico communities are constructed and used in exactly the same way, that is, they are evenly distributed communities consisting out of the same number of cells and made up out of the same bacterial taxa. Classifiers are trained on 70% of the data and evaluated on the opposite 30% data.

	Comb. 1	Comb. 2	Comb. 3
AUC LDA*	0.64	0.82	0.96
AUC LDA	0.62	1.0	0.93
acc LDA*	0.62	0.77	0.92
acc LDA	0.59	0.99	0.91
AUC RF*	0.82	0.94	1.0
AUC RF	0.70	1.0	0.99
acc RF*	0.75	0.87	0.99
acc RF	0.64	1.0	0.97

We note that although the performances are similar for Comb. 3, this is not the case for Comb. 1 and 2. Whereas the performances for Comb. 1 initially reported higher, the performances for Comb. 2 initially reported lower. This could explain why the RMSE for the in vitro analysis for Comb. 2 and 3. has similar precision. However, this implies that although our approach is fruitful to analyze synthetic communities, performances are not yet reproducible when axenic cultures are characterized by FCM at different time points.

### Evaluation of higher complexity in silico communities

**Fig 4:**
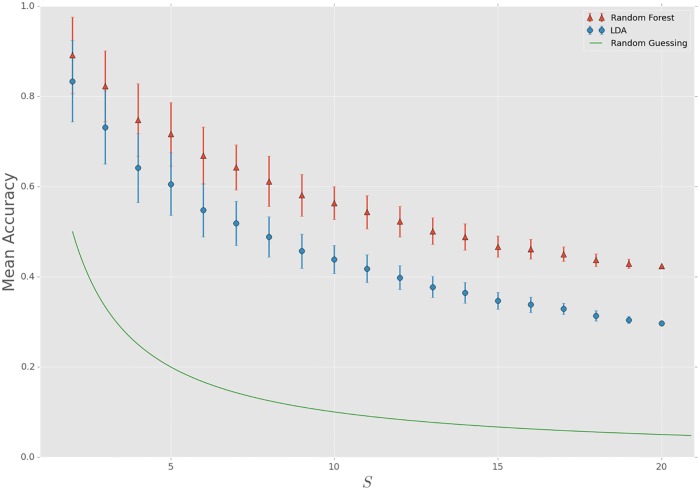
Classifier performances using LDA and Random Forests for increasing population richness. Mean accuracy along with a 68%-CI is displayed, resulting from an analysis using LDA and Random Forests for 150 randomly chosen in silico communities for *S* = 2, …, 18 (for *S* = 19 and *S* = 20 this number is 20 and 1 respectively); every in silico community is evenly distributed, sampling 5,000 cells per population. The accuracy has been calculated on a 30% held-out test set, after which the mean accuracy is calculated for the ensemble of silico communities for every increment of *S*.

In order to explore to what extent single-cell predictions can be made when we increase the population richness, we created in silico communities in a *multiclass* setting. We used the same approach as in the binary setting, but now we let *S* vary from 2, …, 20. To keep it computationally feasible we chose 150 different in silico communities at random for every increment in *S* (except for *S* = 19 and *S* = 20, where we only have 20 and 1 different combinations respectively at our disposal). To quantify our results we calculated the mean accuracy for every *S*; results are displayed in Fig 4.

For all values of *S* our approach is able to make single-cell predictions significantly better than random guessing. As *S* increases, both the mean accuracy and the size of the confidence interval decreases. As the richness increases, the degree in overlap between populations in the multiparametric ‘FCM-space’ starts growing accordingly. Therefore it is harder for classifiers to make a distinction between populations, which results in performances that are lower and more centered.

The difference in performance between the two classifiers increases as *S* increases. This means that for communities with a low richness (*S* = 2, 3) LDA might provide a sufficient method to make single-cell predictions, but as *S* increases Random Forest will be a better option for most communities. This also implies that although for low *S* a linear combination of variables already discriminates populations quite well, predictions can be improved by resorting to classifiers which have the possibility of detecting non-linear relations between variables.

## Discussion

Using the concept of in silico communities, we are able to use supervised machine learning techniques to taxonomically identify bacterial cells up to high accuracies based on FCM data. We note that this approach has not yet been adopted to analyze the composition of synthetic bacterial communities. A possible reason for this is the lack of incorporating these methods in standard FCM software [27].

Using a full combination of fluorescence and scatter signals, we demonstrated that using ‘off-the-shelf’ classifiers without further data manipulation already results in acceptable to high performances for low population richness. Compared to previous research, we note that Rajwa et al. were not able to use LDA in order to make proper single-cell predictions [21]. While they were limited to the combination of scatter signals, we also incorporated fluorescence parameters in our analysis, thereby improving the amount of single-cell information that is acquired. In our study, we applied a single staining approach; there exists, however, a wide array of fluorescent viability markers, all of which may harbor additional single-cell information [28, 29]. Preliminary observations have already revealed the differential behavior of bacterial taxa to these staining protocols [28]. As the number of dimensions and the amount of fluorochromes describing a single-cell is increasing, we expect our approach only to gain in utility in the near future. A natural extension of this research would be to find the optimal classification method to analyze FCM data, which should be extensible to a multiclass setting; a number of possibilities exist, ranging from binary classifiers which are naturally extendable to a multiclass setting or a combination of binary classifiers using a *one-versus-one (OVO)* or *one-versus-all (OVA)* approach [30].

Although it has been briefly mentioned in literature that an in silico community can be representative for its in vitro counterpart [20], there is a lack of rigorous studies proving this observation. We feel that this question has been answered more thoroughly by systematically retrieving the composition of synthetic communities across an abundance gradient. The results imply that in silico communities form a valid representation of synthetic communities. However, although the performance of classifiers gives a good indication to what extent populations are distinguishable, it is not always possible to reproduce the classifier performance in different experiments. This observation can be attributed to two sources of variation, namely *technical* variability and *biological* variability. It has been shown that both sources give rise to heterogeneity in FCM data when studying bacterial axenic cultures [24], although it is difficult to distinguish between one another [31].

Technical variability has been suggested to arise from the time-dependent bleaching and leaking of fluorochrome molecules [32]. Its effect on the classifier performance becomes clear when conducting an in silico performance evaluation using individual replicates (instead of pooling them, as they are measured in duplicate). Creating two sets of replicates for *S* = 2, A & B, we see that for a significant number of combinations the difference in classifier performance is noteworthy, with a mean difference of 2% and a standard deviation of 11%. For clarity, we added the Random Forest performances (A, B and pooled) for all in silico communities in S1 File. However, referring to the results of the in vitro analysis, we note that pooling replicate samples compensates this experimental bias and is sufficient to retrieve the composition of an in vitro community. In order to reduce technical variability as much as possible, we do suggest to include a higher number of replicates for future experiments. To find this number, the strategy of Davis et al. can be followed [33], which suggests that less than five replicates (but more than two) are sufficient for most experiments.

Biological variability is another and perhaps more important factor to take into account when analyzing microbial communities with FCM. Vives-Rego et al. hypothesize that biological variability in FCM stems from cell size diversity and cell cycle variations [24]. In this study we tried to control for this variability by focusing on cultures in the stationary growth phase, so that we could directly compare the performance of the analysis. Yet overall, the multitude of biological processes that result in single cell physiological variation still remain largely undefined [34]. Results of this research comply with motivations that FCM can be used to further characterize bacterial heterogeneity and physiology [35–37], for which a holistic approach has been proposed [13].

To do so, a more comprehensive protocol is required to make our in silico approach fully operational, a need which has been pointed out before [38]. This protocol includes further improvement of data-analysis techniques, such as automated denoising, but also a more developed methodology to reduce sources of variability, both of instrumental and biological origin. However, we believe that the combined approach of microbial flow cytometry and machine learning supports this endeavor, and this will be the main focus of further research.

For now, in silico communities can already be exploited for various purposes. For environments where limited physiological variation in the axenic populations is expected, or where the in silico populations have been defined for all possible physiological states, our approach can be used to retrieve the community composition for low-complexity microbial communities. Furthermore, by using evaluation tools for classifiers such as the accuracy or the AUC, one can quantify which populations are distinguishable and which are not. One intuitive tool which is extensible to a multiclass setting, is the use of a confusion or misidentification matrix. This allows one to inspect which populations are likely to overlap and which are not; an example is given in S1 Fig.

Secondly, as we have shown that in silico communities form a viable representation of their in vitro counterparts, we are allowed to extrapolate properties of in silico communities to in vitro microbial communities. This means that in silico communities can be used as a stand-in for in vitro communities, enabling us to use them to develop new data-driven techniques, which will ultimately lead to novel applications for microbial FCM.

## Materials and Methods

### In silico communities

An in silico community consists out of an aggregation of data coming from axenic cultures, which are being measured separately through FCM. As we have twenty axenic cultures at our disposal, the population richness (*S*) of an in silico community varies from *S* = 2, …, 20.

### Learning in silico communities

Each bacterial population was sampled in equal size. We randomly subsampled *N*_ax_ = 5,000 cells per axenic culture. This means that an in silico community consists out of *N*_tot_ = *S* × *N*_ax_ cells. We used 70% of an in silico community to train a classifier, this is the *training* set; the other 30% was held-out and used to evaluate the performance of a classifier, the *test* set.

For *S* = 2 we evaluated the performance of LDA and the Random Forest classifier for all possible pairwise combinations, which is 190. For increasing *S*, i.e. the multiclass setting, we evaluated the performance for 150 randomly chosen combinations for *S* = 2, …, 18 (for *S* = 19 and *S* = 20 we chose the maximum number of combinations, which is 20 and 1 respectively), in order to keep it computationally feasible. For every increment of *S* we calculated the mean accuracy, averaging the accuracies for all 150 randomly chosen in silico communities.

### Learning in silico communities to predict the abundance gradient

We used the concept of an *abundance gradient* to prove that properties of in silico communities can be used for the identification of their in vitro counterparts. An abundance gradient consists out of a set of microbial communities where populations have been mixed in varying abundances. We created an abundance gradient both in silico and in vitro for three combinations of two populations, with abundances ranging from 1% to 99% for the one population and vice versa for the other. This was possible as we measured the axenic cultures separately through FCM beforehand. We chose three different combinations of two populations to create abundance gradients, combinations which initially reported a low, medium and high performance respectively, based on the performance of the Random Forest classifier for *S* = 2. For every community in an abundance gradient we sampled 10,000 cells (both in silico and in vitro), except for one in vitro community of Comb. 3, for which we were not able to register enough cells (see further on).

We trained a classifier on an evenly sampled in silico community to predict the label of individual cells for all communities in an abundance gradient. We sampled *N*_ax_ = 5,000 cells per bacterial population to create the in silico community upon which we trained our classifier; as the abundance gradient acts as our test set, we trained our classifier on the full in silico community. Note that we have cultivated and measured new axenic cultures in order to create both the in silico and in vitro abundance gradients.

### Datasets

#### Dataset 1: axenic cultures

Twenty bacterial populations were gathered from publicly available culture collections, of which a full list can be found in Table 4. Populations with reference numbers LMG, R, DSMZ and UFZ originate from the collection of LM-UGent, Belgian co-ordinated collection of microorganisms BCCM/LMG (www.bccm.belspo.be), Leibniz Institute DSMZ (www.dsmz.de) and Helmholtz Centre for Environmental Research (www.ufz.de/index.php?en=13354), respectively. For cultivation, all bacteria were grown on rich, solid lysogeny broth medium (LB; Carl Roth, Germany). A single colony was picked and cultivated for 48h in liquid LB medium. Finally, fresh LB medium was inoculated with 10% (v/v) inoculum and incubated for 24h; all samples were measured in duplicate. A bivariate scatterplot (FL1-H vs. FL3-H) after denoising can be found for every culture (S2 Fig); >10,000 cells were registered for each measurement.

**Table 4:** List of axenic cultures measured individually through FCM.

Bacterial population	Culture collection reference
*Agrobacter rhizogenes*	UFZ [7]
*Bacillus subtilis*	LMG 7135
*Burkholderia ambifaria*	LMG 19182
*Citrobacter freundii*	DSMZ 15979
*Cupriavidus necator*	LMG 1201
*Cupriavidus pinatubonensis*	LMG 1197
*Edwardsialla ictaluri*	LMG 7860
*Enterobacter aerogenes*	DSMZ 30053
*Escherichia coli*	DSMZ 2840
*Janthinobacterium sp. B3*	UFZ [7]
*Klebsiella oxytoca*	LMG 3055
*Lactobacillus plantarum*	LMG 9211
*Micrococcus luteus*	UFZ [7]
*Pseudomonas fluorescens*	R 23898
*Pseudomonas putida*	R 17801
*Rhizobium radiobacter*	LMG 287
*Shewanella oneidensis*	LMG 19005
*Sphingomonas aromaticivorans*	LMG 18303
*Streptococcus salivarius*	LMG 11489
*Zymomonas mobilis subsp. mobilis*	LMG 460

#### Dataset 2: abundance gradients

To create abundance gradients, we chose three combinations (Comb.) of two bacterial populations based on Random Forest performances calculated during the in silico analysis of dataset 1 (Table 5). We measured the exact cell densities of both bacterial cultures through FCM, and used them to calculate the required volumetric proportions to construct a relative abundance gradient of 1%, 5%, 10%, 20%, 30%, 40%, 50%, 60%, 70%, 80%, 90%, 95% and 99%. After 24h of growth, cells were diluted in 0.2 μm filtered PBS for FCM measurement. Based on these cell densities both cultures were diluted in 0.2 μm filtered PBS to an equal cell density of approximately 10^8^ cells mL^-1^, which was verified through an additional FCM measurement. The equal density suspensions were then mixed in the required proportions to final volumes of 500 μL. All samples were subsequently measured in triplicate through FCM (for Comb. 2 the axenic cultures were measured in quadruplicate). >10,000 cells were registered for each measurement, except for Comb. 3 where we registered 3.084 cells for 1% of *S. oneidensis*.

**Table 5:** Three different combinations of bacterial populations used to create abundance gradients.

Comb.	Population 1	Population 2	initial RF accuracy
1	*P. fluorescens*	*P. Putida*	low (0.75)
2	*A. rhizogenes*	*Janthinobacterium sp. B3*	medium (0.87)
3	*M. luteus*	*S. oneidensis*	high (0.99)

As we measured the populations separately beforehand through FCM, we were also able to construct an abundance gradient in silico, by sampling communities according to the same relative abundances as described above.

#### FCM analysis

Samples were diluted until an approximate cell density of 10^6^ cells mL^-1^ in 0.2 *μ*m filtered buffer solution was reached (PBS; 6.8 g L^-1^ KH_2_PO_4_, 8.8 g L^-1^ KH_2_PO_4_ and 8.5 g L^-1^ NaCl) and stained with a final concentration of 1% (v/v) nucleic acid stain SYBR^®^ Green I (100x concentrate in 0.2 μm filtered dimethyl sulfoxide). Samples were incubated for 20 minutes in the dark at 37°C. FCM measurements were performed on a C6 Accuri flow cytometer (BD Biosciences, Belgium) equipped with four fluorescence detectors (530/30 nm, 585/40 nm, 670 nm LP, 675/25 nm), two scatter detectors and a 20mW 488nm laser. This results in a multiparametric description of each cell consisting out of twelve variables (FL1-A, FL1-H, FL2-A, FL2-H, FL3-A, FL3-H, FL4-A, FL4-H, FSC-A, FSC-H, SSC-A and SSC-H).

#### Data preprocessing

The FCM data were denoised from (in)organic and instrument noise by means of a reproducible digital gating strategy in the arcsinh(*x*) transformed FL1-FL3 bivariate space, following the guidelines by Hammes et al. [39] and Prest et al. [40]. This filtering strategy was verified by negative controls (non-stained samples) and kept fixed for all samples of the same axenic culture and within each abundance gradient. An example of the gating stratey has been given for the 40%/60% abundance files for all three combinations used to create abundance gradients (S3 Fig). Filtered data files were exported as individual FCS files with the write.FCS function. The cell densities for each individually measured taxon were calculated by the filter function; both functions are available from the flowCore package (v.1.38.1). As axenic cultures and synthetic communities have been measured in a number of replicates, we pooled these replicates together first, before subsampling cells to create our in silico and in vitro communities we use for analysis.

### Classifiers

#### Linear Discriminant Analysis

Linear Discriminant Analysis (LDA) is a linear classifier which tries to find the optimal linear combination of features in order to separate objects or classes. It assumes the data are distributed according to a Gaussian distribution. It has no hyperparameters to tune and is able to handle problems in the multiclass setting in a natural way. For more information, see [41] or chapter 4.3 in [42].

#### Random Forests

A Random Forest classifier is an example of an *ensemble method*, a method in which various classifiers are trained and in which a majority vote is taken to predict the outcome of an unknown sample. In this case the ensemble consists out of decorrelated unpruned trees grown on bootstrap samples. The trees are decorrelated because at every split only a random subset of the total number of *K* variables is available (*K* = 12). This results in a decrease in variance for only a slight increase in bias, hence lowering the overall classification error. For more information see [43] or chapter 15 in [42].

We grew 200 trees when training a Random Forest and chose the gini criterion when making a split. We note that there is no need to tune the number of features that are available to choose from when making a split. We applied the preset $\sqrt{K}$, which resulted in (near-)optimal results, in accordance with [44]. This has been verified by comparing the performance for twenty randomly chosen in silico communities for *S* = 2, …, 19 using the preset $\sqrt{K}$ as opposed to determining this value by 10-fold cross-validation. The increase in accuracy never reported higher than 0.7%.

### Performance measurement

We used various performance metrics in order to evaluate our methodology. We evaluated the in silico analysis in terms of the *accuracy* and the *area under the receiving operating characteristic curve* (AUC). The in vitro analysis is expressed in terms of the *root mean squared error* (RMSE).

#### Accuracy

The accuracy can be defined in the following way:

$$\text{accuracy}=\frac{1}{N}\sum_{i=1}^{N}1\left({\hat{y}}_{i}=y_{i}\right),$$ (1)

where *N* denotes the total number of elements to predict, $\hat{y}$ the predicted label of an element, *y* the true label and **1** the indicator function, which returns the value of 1 when its argument is true and 0 otherwise. It can also be expressed in terms of the true positives (tp), the number of correctly predicted elements belonging to a certain class *j*, true negatives (tn) the number of correctly predicted elements not belonging to class *j*, false positives (fp), the number of incorrectly predicted elements belonging to class *j* and false negatives (fn), the number incorrectly predicted elements not belonging to class *j*. In this setting, the accuracy can be written as:

$$\text{accuracy}=\frac{\sum_{j=1}^{S}\frac{tp_{j}+tn_{j}}{tp_{j}+tn_{j}+fp_{j}+fn_{j}}}{S},$$ (2)

where *S* denotes the total number of classes.

#### Area under the receiver operating characteristic curve

The AUC measures the area under the *receiving operating characteristic* (ROC) curve and can be used as a performance measurement for a binary classifier [45, 46]. The ROC curve is a curve which is constructed by calculating the tp rate versus the fp rate for various thresholds. These thresholds can be determined for classifiers which assign probabilities to predictions; this is the case for both LDA (applying Bayes’ theorem) and for Random Forests (applying a majority vote for the ensemble of trees).

Calculating this area results in a number between 0 and 1; the higher this number, the better the performance of a classifier. The AUC can be interpreted as the probability that a classifier will rank a randomly chosen positive higher than a randomly chosen negative. Using the AUC has a number of favorable properties. Most notable are the fact that it gives an indication of how well separated the positive and negative class are and that it is insensitive to prior skewness concerning class distributions.

#### Root mean squared error

Expressing the known relative abundance as *p*, opposed to the predicted $\hat{p}$, the RMSE becomes:

$$\text{RMSE}=\sqrt{\frac{\sum_{i=1}^{n}{\left(p_{i}-{\hat{p}}_{i}\right)}^{2}}{n}},$$ (3)

with *n* being the total number of bacterial communities constituting an abundance gradient. Therefore when the set of predictions are close to the ground truth, the RMSE lies close to zero.

#### Confusion matrix

A confusion matrix is a tool which helps to describe the performance of a classifier. It reports the tp, tn, fp and fn, and is naturally extendable to a multiclass setting. In this way one can inspect to what extent a classifier ‘confuses’ certain labels of classes. An example for the binary setting is given in Table 6.

**Table 6:** Confusion matrix for a binary setting.

		Predicted	
Actual	label	0	1
	0	tp	fn
	1	fp	tn

Applied to the use of in silico communities, one is able to inspect which populations are easily separated by a classifier and which populations have a similar FCM fingerprint.

### Computational Tools

#### Code availability

Our code has been made available on github: https://github.com/prubbens/InSilicoFlow.

#### Data availability

Our data has been made freely available in .fcs format on the FlowRepository database [47], and can be found using the following identifiers:

- Axenic cultures: FR-FCM-ZZSH.
- Abundance gradients: FR-FCM-ZZSG.

#### flowCore

The data has been preprocessed and exported using flowCore, a package of computational Tools written in R for the analysis of FCM data [48].

#### Scikit-learn

Scikit-learn is an open-source library of various machine learning methods, which can be used in Python [49]. We used its implementation to perform LDA and Random Forests, to calculate the AUC and accuracy, and to perform cross-validation.

## Appendix: Alpha diversity analysis

Knowing the relative abundance of a population, we can determine the Hill number of order one, i.e., the first alpha diversity metric, denoted as *D*_1_ [26]. This index is well established in the context of synthetic ecology and can be used both in a binary and a multiclass setting. It is defined according to the following formula, where *p*_*i*_ denotes the relative abundance of population *i*:

$$D_{1}=\exp\left(-\sum_{i=1}^{S}p_{i}\ln p_{i}\right),$$

An additional advantage using a diversity index such as *D*_1_ is that we can combine uncertainties for the relative abundances. As it has been established that the instrumental variation *ϵ*_*I*_ for FCM is less than 5% considering cell counts [25], we can calculate the variance for the relative abundance $\sigma_{p}^{2}$. As *p* can be calculated from a division of two cell counts, $\sigma_{p}^{2}$ equals:

$$\sigma_{p}^{2}=2\epsilon_{I}^{2}\times p^{2}$$

We can combine the variances for *p*_*i*_ (as *p*_*i*_ can be determined independently) to determine the variance for *D*_1_ in the following way:

$$\sigma_{D_{1}}^{2}=D_{1}^{2}\sum_{i=1}^{S}{\left(\ln p_{i}+1\right)}^{2}\sigma_{p_{i}}^{2}$$

For *S* = 2 (as *p*_2_ = 1−*p*_1_), this formula can be expressed in terms of the relative abundance of the first population *p*_1_ as follows, combining the two formulas above:

$$\sigma_{D_{1}}^{2}=2\epsilon_{I}^{2}D_{1}^{2}\left[p_{1}^{2}{\left(\ln\left(p_{1}\right)+1\right)}^{2}+{\left(1-p_{1}\right)}^{2}{\left(\ln\left(1-p_{1}\right)+1\right)}^{2}\right]$$

Using this information, we calculated *D*_1_ for every community constituting the abundance gradient, and additionally, we gained a confidence interval within which our in vitro constructed *D*_1_ should lie, setting *ϵ*_*I*_ = 0.05; this information is visualized in S4 Fig.

## Supporting Information

S1 File Random Forest performances for all in silico communities for *S* = 2. (XLSX)

S1 Fig Confusion matrix. Example of a confusion matrix calculated on a 30% held-out test set for an in silico community with *S* = 5. Every element of the matrix *m*_*ij*_ gives the fraction of the population *i* that is predicted as population *j*.
(TIF)

S2 Fig Scatterplots of all individual bacterial populations after denoising. Flow cytometric characterization of 20 bacterial taxa. Each point represents one single cell characterized by two fluorescence parameters (FL1-H and FL3-H). The data were denoised from (in)organic noise based on a reproducible digital gating strategy (explained above) and was adjusted for each taxon.
(TIF)

S3 Fig Filtering strategies. **A** Comb. 1: *P. putida—P. fluorescens*; **B** Comb. 2: *A. rhizogenes—Janthinobacterium sp. B3*; **C** Comb. 3: *S. oneidensis—M. luteus*. Data filtering strategy for the FCM data for each abundance gradient based on a reproducible digital gating strategy (explained above); data points outside the filter represent (in)organic noise. Examples are given for the 40%-60% abundance files.
(TIF)

S4 Fig Predicted abundance gradients expressed in terms of alpha diversity *D*_1_. **AB** Comb. 1: *P. putida—P. fluorescens*; **CD** Comb. 2:*A. rhizogenes—Janthinobacterium sp. B3*; **EF** Comb. 3: *S. oneidensis—M. luteus*. Both predicted and target *D*_1_ is plotted against the relative abundance of the first population for every combination, both for the in silico (left panel) and in vitro (right panel) abundance gradients; the 68%-CI and 95%-CI for the in vitro gradients are also visualized, determined as described in Appendix: Alpha diversity analysis.
(TIF)
