# Supplementary figures and images for: Flow Cytometric Single-Cell Identification of Populations in Synthetic Bacterial Communities
Source: PLoS One. 2017 Jan 25;12(1):e0169754. doi: 10.1371/journal.pone.0169754 (PMC5266259; doi:10.1371/journal.pone.0169754)

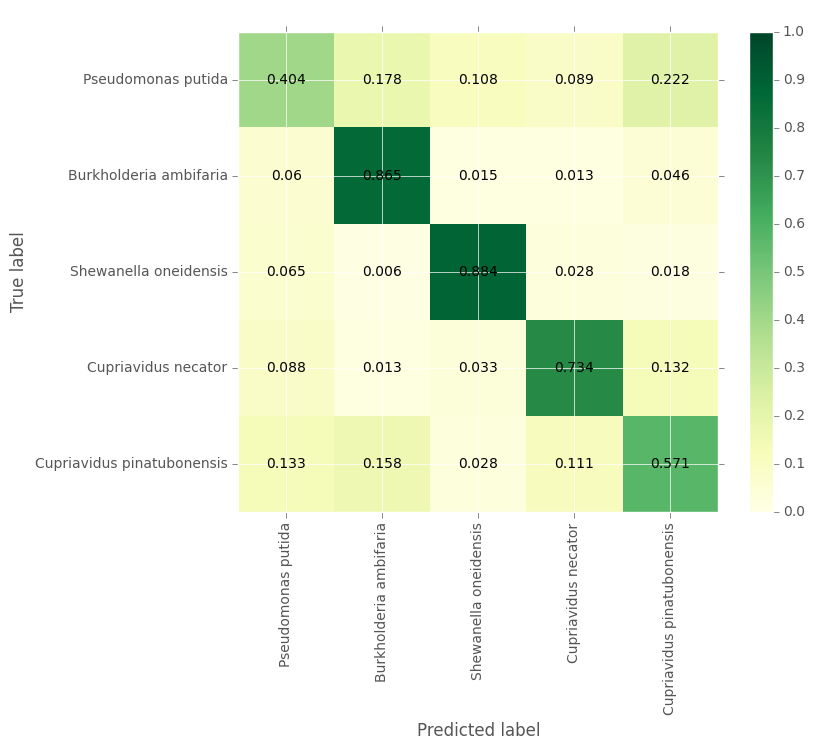

Supplement: S1 Fig — Example of a confusion matrix calculated on a 30% held-out test set for an in silico community with S = 5. Every element of the matrix mij gives the fraction of the population i that is predicted as population j. (TIF) [file pone.0169754.s002.tif]

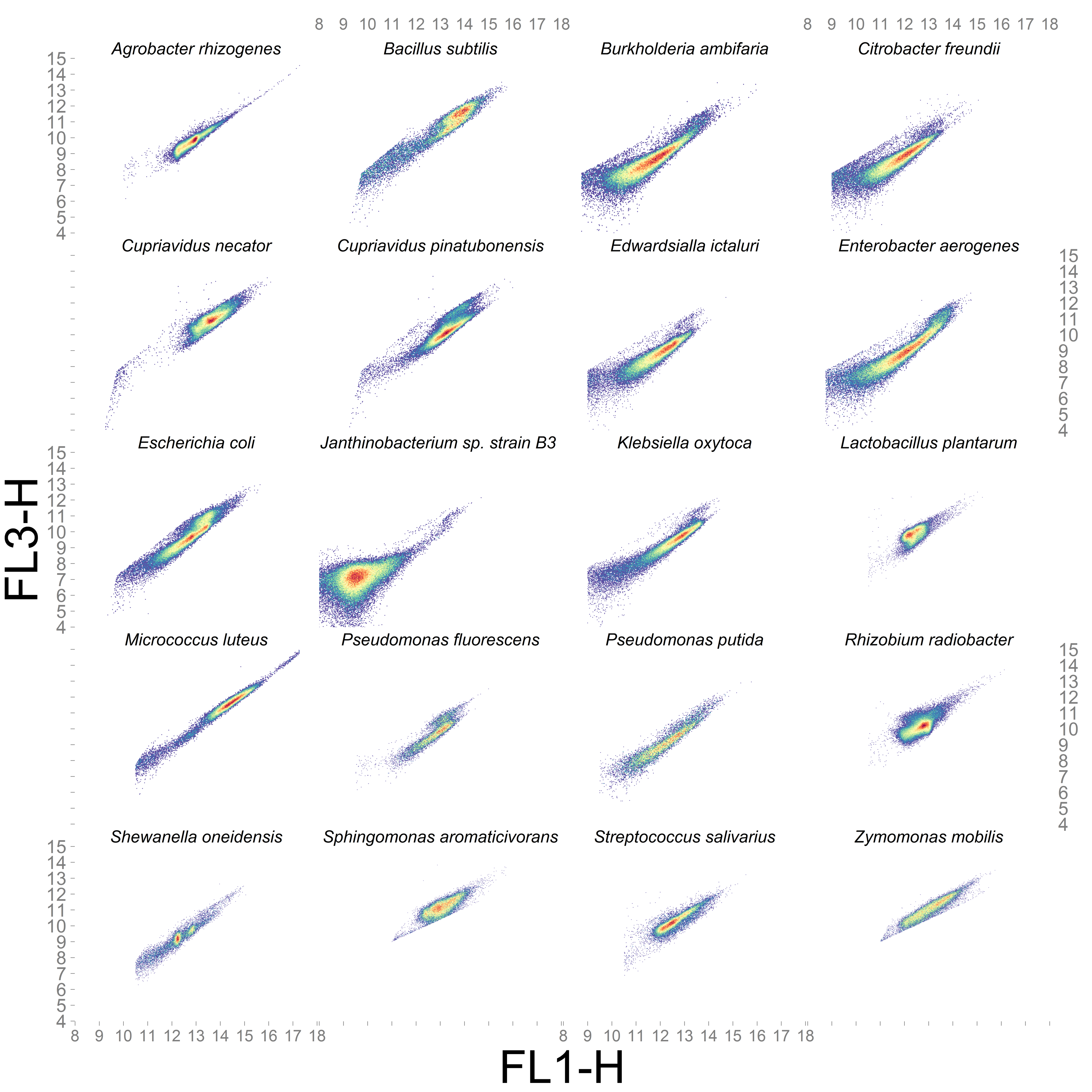

Supplement: S2 Fig — Flow cytometric characterization of 20 bacterial taxa. Each point represents one single cell characterized by two fluorescence parameters (FL1-H and FL3-H). The data were denoised from (in)organic noise based on a reproducible digital gating strategy (explained above) and was adjusted for each taxon. (TIF) [file pone.0169754.s003.tif]

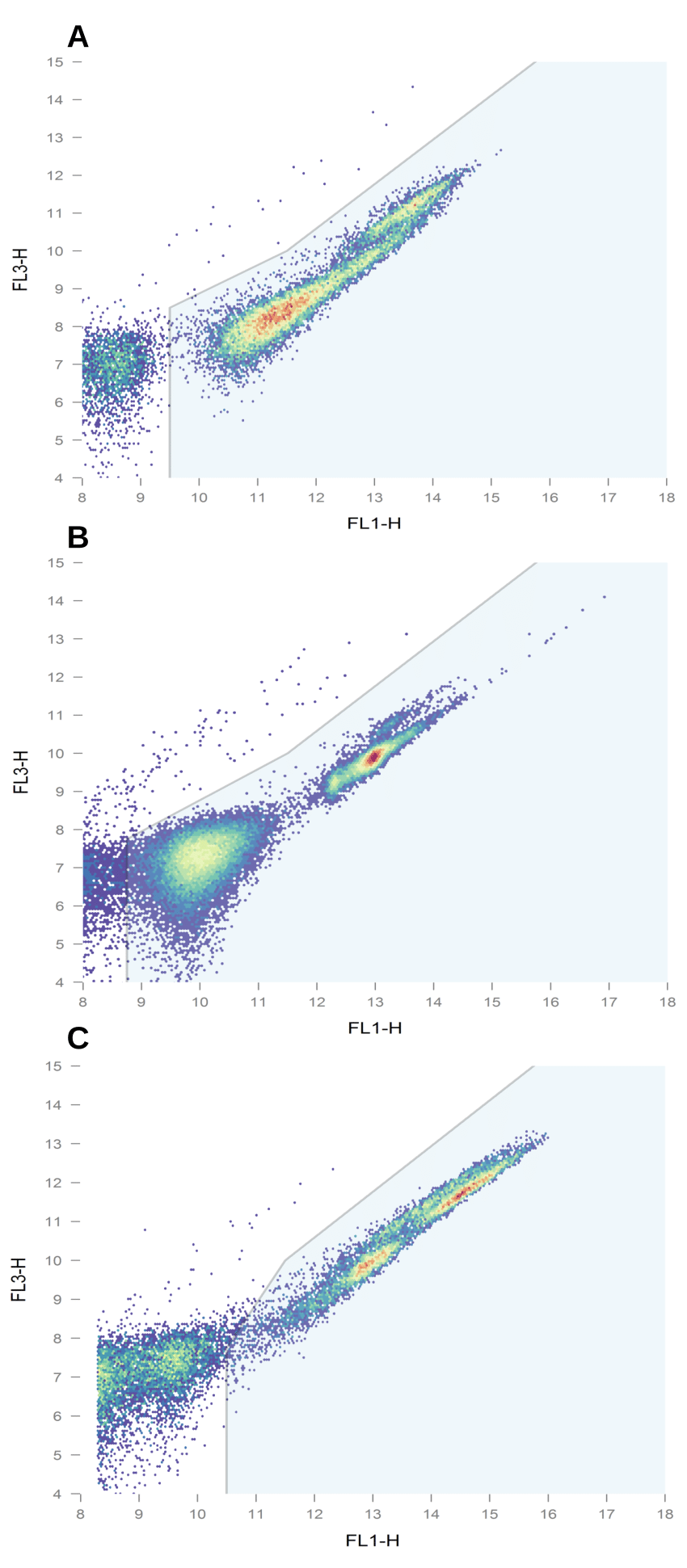

Supplement: S3 Fig — A Comb. 1: P. putida—P. fluorescens; B Comb. 2: A. rhizogenes—Janthinobacterium sp. B3; C Comb. 3: S. oneidensis—M. luteus. Data filtering strategy for the FCM data for each abundance gradient based on a reproducible digital gating strategy (explained above); data points outside the filter represent (in)organic noise. Examples are given for the 40%-60% abundance files. (TIF) [file pone.0169754.s004.tif]

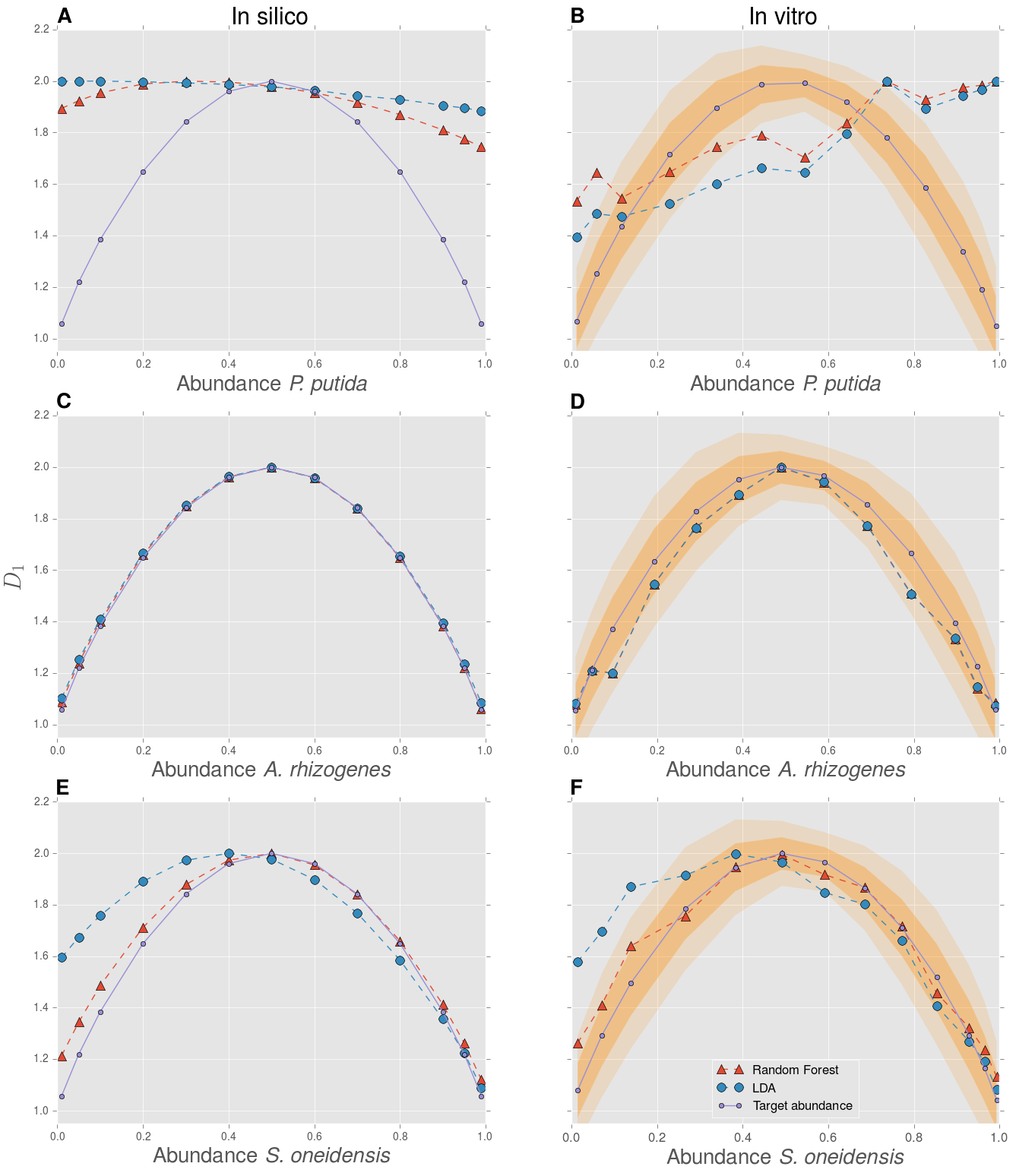

Supplement: S4 Fig — AB Comb. 1: P. putida—P. fluorescens; CD Comb. 2:A. rhizogenes—Janthinobacterium sp. B3; EF Comb. 3: S. oneidensis—M. luteus. Both predicted and target D1 is plotted against the relative abundance of the first population for every combination, both for the in silico (left panel) and in vitro (right panel) abundance gradients; the 68%-CI and 95%-CI for the in vitro gradients are also visualized, determined as described in Appendix: Alpha diversity analysis. (TIF) [file pone.0169754.s005.tif]
